# *LAHMA*: structure analysis through local annotation of homology-matched amino acids

**DOI:** 10.1107/S2059798320014473

**Published:** 2021-01-01

**Authors:** Bart van Beusekom, George Damaskos, Maarten L. Hekkelman, Fernando Salgado-Polo, Yoshitaka Hiruma, Anastassis Perrakis, Robbie P. Joosten

**Affiliations:** a Oncode Institute and Division of Biochemistry, Netherlands Cancer Institute, Plesmanlaan 121, 1066 CX Amsterdam, The Netherlands

**Keywords:** protein structure, homology, webserver, structure analysis, *LAHMA*

## Abstract

Comparison of homologous structure models is a key step in analyzing protein structure. With a wealth of homologous structures, comparison becomes a tedious process, and often only a small (user-biased) selection of data is used. A multitude of structural superposition algorithms are then typically used to visualize the structures together in 3D and to compare them. Here, the *Local Annotation of Homology-Matched Amino acids* (*LAHMA*) website (https://lahma.pdb-redo.eu) is presented, which compares any structure model with all of its close homologs from the PDB-REDO databank. *LAHMA* displays structural features in sequence space, allowing users to uncover differences between homologous structure models that can be analyzed for their relevance to chemistry or biology. *LAHMA* visualizes numerous structural features, also allowing one-click comparison of structure-quality plots (for example the Ramachandran plot) and ‘in-browser’ structural visualization of 3D models.

## Abstract 2

The *LAHMA* web server for structural analysis of homologous proteins is presented.

## 1. Introduction   

The Protein Data Bank (PDB; Bernstein *et al.*, 1977 ▸; Burley *et al.*, 2019 ▸) contains an ever-increasing wealth of homologous structural data. As of June 2019, the median number of homologous protein chains with a sequence identity of 70% is 28, while the average is 145. It is difficult to visualize such a wealth of data efficiently. Structure models are compared by structural superposition, which can be performed by a large repertoire of algorithms that place emphasis on different features of the structure models. Effective comparison typically allows only a few structure models before the view becomes too crowded to be informative. Furthermore, differences are easily overlooked or difficult to assess in a visual inspection by means of superposition, especially if they are subtle or involve large (local) changes that make alignment particularly challenging or if there is a very large set of homologs available for inspection. The latter is, for instance, the case in the calcium-binding sites of calmodulin, which has 360 close homologs in the PDB.

Comparing the models while avoiding the superposition step, which implicitly requires many choices as to the type of superposition algorithm and its parameters (for example, when considering how to compare a multi-domain protein with relative domain movement), has advantages (Nicholls *et al.*, 2014 ▸). Also, automating this process has the potential to remove user bias and make the task easier and less error-prone. This requires breaking the comparison of structure models down to the comparison of specific structural features, such as dihedral angles. While these comparisons at first look less sophisticated than a visual comparison of superposed structure models, the number of comparisons that can be performed is far larger. In addition, when studying the distribution of model features, outliers which would have otherwise gone unnoticed can be detected. Also, multimodal distributions of model parameters can be detected more easily. Therefore, comparison of homologous structure models can uncover potentially interesting and/or problematic sites of a structure model that would otherwise have gone unnoticed. Hence, inspecting protein structure models and their families in an automated fashion could be more informative and less time-consuming, provided that suitable tools are available.

A few tools have already been made available to automatically compare protein structure models at the residue level: *Phenix Structure Comparison* (Moriarty *et al.*, 2018 ▸) and 2*StrucCompare* (Drew & Janes, 2019 ▸). Both offer a selection of comparison features and require users to manually identify homologs. 2*StrucCompare* limits the comparison to two structure models and thus does not harness the power of comparing information on all homologs to extract statistically significant outliers. *Phenix Structure Comparison* is mostly designed to deal with (almost) sequence-identical homologs. We thus considered a tool offering automated all-homologs, all-features comparison within homologous model families.

Here, we present *Local Annotation of Homology-Matched Amino acids* (*LAHMA*), a web server that compares a protein structure model with all homologous protein chains in the PDB-REDO databank (Joosten *et al.*, 2012 ▸). Importantly, this builds on our previous work on using homology-derived restraints for refinement (van Beusekom, Touw *et al.*, 2018 ▸): thus, differences that persist in the PDB-REDO databank after imposing homology restraints are more likely to be genuine. The website shows both the general quality of a protein structure model and its quality compared with its homologs in sequence space. *LAHMA* provides the users with a wealth of information aggregating all of the structure features that we could think of as being useful: for instance, it determines which Ramachandran angles or rotamers are outliers with respect to homologous chains, which residues have a relatively poor density fit, which residues are post-translationally modified in other structure models, and many other features. A simple click on a residue of interest in the sequence takes the user to a screen where all of the metrics of the query structure are compared with all homologs, and another click leads to a 3D visualization within the browser window. For each PDB-REDO entry, the data are pre-computed and thus quickly visualized, but it is also possible for the user to upload PDB files and compare them with all homologs found in the databank. Thus, *LAHMA* can be used to easily uncover potentially erroneous features in a structure model, but more importantly it can also be used to uncover interesting differences between homologous structure models that have previously been overlooked.

## 2. Methods   

### 2.1. Database setup   

A MariaDB database server (https://mariadb.org/) was set up to store data for all structure models in the PDB-REDO databank and was mined by the C++ program *annotator* to allow visualization in *LAHMA*. A scheme of the LahmaDB database is provided in Supplementary Fig. S1. The database contains 11 tables. The Residue, ResData, Parameter, Contact and Ligand tables provide all of the protein annotation information; the data in these tables is explained in more detail below. The Entry table simply contains the PDB identifier, resolution, space group, species and *R*_free_, the Homol­map table contains all of the information required to map homologous residues onto one another, the Warning table contains warnings that can be shown to users on the website (for example, ‘No protein detected’ for DNA/RNA entries), the NCSInfo table contains information on which protein chains in which entries are NCS-related (for smarter visualization on the website) and, finally, the RedoChanges and RedoParameter tables contain information about the changes in the PDB-REDO structure model compared with the PDB version of the model. The changes are shown on the residue webpage: if something is an outlier, it is relevant to determine whether this was caused by application of the fully automated *PDB-REDO* pipeline. This could either mean that something was unobserved before or that *PDB-REDO* has wrongly changed a structural feature. The determined features are changes in rotamers, hydrogen-bonding flips of Asn/Gln/His residues, newly introduced *cis* and *trans* conformations, addition or removal of distorted ω angles, and removed or added amino-acid residues, water molecules or other compounds, side chains and C-terminal O atoms.

The structural data and features that are stored for every residue are shown in Table 1 ▸. The computation of these metrics is discussed in Section 2.2.

### 2.2. Homologous residue data   

We retrieve homologs by performing a *BLAST* (Altschul *et al.*, 1990 ▸) search against a local sequence database of all PDB-REDO entries. Only close homologs with a sequence identity of 70% and an *E* value of less than 10^−3^ are used in order to ensure that structures can be expected to be similar enough for detailed comparison. The 70% cutoff was determined empirically in a previous study on homologous hydrogen-bond conservation (van Beusekom, Touw *et al.*, 2018 ▸). For each *LAHMA* entry, *annotator* maps homologous residues onto the residues of the structure of interest based on the local alignment from *BLAST*.

For each amino-acid residue and all ligand molecules (except water), the PDB identifier, chain identifier, residue type, residue number, insertion code and (only for amino acids) whether or not it is ordered (*i.e.* modeled in the PDB-REDO entry) are stored. This section explains in detail how each of the parameters is collected.

#### 2.2.1. Ramachandran and rotamer torsion angles   

Both the Ramachandran *Z*-score and the rotamer *Z*-score are calculated based on a reimplementation of the algorithm from *WHAT_CHECK* (Hooft *et al.*, 1997 ▸) as has been described previously (van Beusekom, Joosten *et al.*, 2018 ▸; Sobolev *et al.*, 2020 ▸).

2D *k*-means classification is then run on the Ramachandran angles (φ and ψ) of the current residue and its homologs. A maximum number *k* of three clusters is allowed and ten trial classifications are run for each *k*. The classification trial with the smallest sum of the squared distances to the cluster centers is selected as the best. The optimal number of clusters is then determined by computing the gap statistic (Tibshirani *et al.*, 2001 ▸). This statistic is a measure of how well the data are clustered for a given number of clusters compared with the classification of random data into the same number of clusters; the number of clusters for which this difference is largest is then picked as the optimal number of clusters. For the calculation of each gap statistic, ten trials of random data are generated to obtain a good estimate of random clustering. Next, clusters that are very close (within 30° or within 60° but also within 1.5σ) are merged. Also, clusters that are very small (less than 5% of the residues or less than 25% but also having at most five members) are merged with the nearest cluster. Minority clusters are identified as clusters that are at least 20 percentage points smaller than the largest cluster. Finally, outliers are determined per cluster: any residue is marked as a strong outlier or a weak outlier if it is more than 5σ or 3σ away from the cluster center, respectively, provided that is at least 40° away from the cluster center.

In the clustering of angles, it is important to take periodicity into account. Therefore, the smallest bounding box around all φ and ψ angles in 2D is determined, taking into account the periodicity of 360°. For instance, if all φ angles range from 160° to 180°, with the exception of one homolog at −175°, the latter is transformed to +185° so that all φ angles are within 25° of one another.

Finally, if the Ramachandran angles of a residue in the structure of interest are outliers or minority conformations then this is stored in the database. We also save the values of Ramachandran angles and all side-chain χ angles to the database in order to compare (and plot) homologous residues later.

#### 2.2.2. Real-space correlation scores   

Firstly, the real-space correlation coefficients (RSCCs) are calculated by *stats* (van Beusekom *et al.*, 2019 ▸). The RSCC values are then converted to *Z*-scores as described previously (Joosten *et al.*, 2014 ▸). Both the RSCCs and *Z*-scores are saved in the database.

#### 2.2.3. Relative *Z*-scores   

The *Z*-scores for Ramachandran angles (Sobolev *et al.*, 2020 ▸), rotamericity and RSCC are also compared across homologs. If a particular residue has, for instance, a low Ramachandran *Z*-score, but the homologous residues also have poor *Z*-scores, it becomes more likely that the protein is in a somewhat strained conformation at this position and less likely that it is wrong.

#### 2.2.4. Rotamer percentages   

Rotamer *Z*-scores measure the quality of a rotamer compared with a large data set of rotamers. A more specific metric can however be obtained by direct comparison of all homologous side chains. The rotamer codes are defined according to the *MolProbity* convention that χ angles around 60° are named *p* (*gauche* plus), those around −60° are named *m* (*gauche* minus) and those around 180° are named *t* (*trans*) (Lovell *et al.*, 2000 ▸). For the torsions around *sp* ^3^–*sp* ^2^ bonds, for example the χ_3_ angle of glutamine, the same approach is used with six intervals. Rotations of 180° in symmetric side chains, for example in tyrosine, are not treated as changes in rotamers. At each position in the structure of interest, the relative frequency of the current rotamer as well as the total number of different rotamers found in the homologs are saved to the database.

#### 2.2.5. *Cis*–*trans* angles   

By far the majority of peptide bonds are in the *trans* conformation, but occasionally these bonds are in the *cis* conformation, most often when adjacent to a proline. Firstly, we calculate the (*i* − 1, *i*) ω angle of each residue and save it to the database. Additionally, every residue is assigned as *cis* (around 0°), *trans* (around 180°) or distorted (more than a 30° deviation from ideal *cis* or *trans*). If the ω angle is distorted, this is immediately saved to the database: this conformation is likely to be wrong regardless of homology. If residues are *cis* or *trans*, the percentage of each conformation is computed. If the conformation of the structure of interest is the same as at least 70% of the homologs, nothing is saved to the database: the conformation is ‘normal’. If the conformation is the same in 70–30% of the homologs, in 30–10% of the homologs or in less than 10% of the homologs, the residue is marked as a variable conformation, a minority conformation or an outlier, respectively. In cases where only few homologs are available, residues are also considered to be outliers if less than 30% of the homologs are in the same conformation, but only if at most two outliers are found in all homologous protein chains. These cutoffs were established empirically during testing of *annotator*.

#### 2.2.6. Symmetry contacts   

The number of symmetry contacts is computed using functionality from the Clipper library (Cowtan, 2003 ▸). A symmetry contact between two residues is defined here as a distance of less than 3.5 Å between any two atoms of a residue pair, one of which is generated by performing symmetry operations. Symmetry copies are generated of all macromolecules and of compounds attached to the protein via LINK records (for instance in the case of *N*-glycosylation). There are no symmetry contacts defined for water molecules, metal ions and other ligands.

#### 2.2.7. Data derived from *DSSP* and *HSSP*   

Two criteria are directly obtained from *DSSP* (Touw *et al.*, 2015 ▸; Kabsch & Sander, 1983 ▸): the secondary structure and the surface accessibility. The secondary structure is defined in *DSSP* as one of the following categories: α-helix, β-strand, 3_10_-helix, π-helix, hydrogen-bonded turn, β-bridge, bend or other. The secondary structure of all homologous residues is also computed using *DSSP*. From this, the percentage of homologs with the same secondary structure as the model of interest is calculated and stored in the database.

The surface accessibility is defined in *DSSP* in Å^2^. To obtain a measure of relative surface accessibility, the data are saved as a percentage of the maximum surface-accessibility values. We generated idealized single-amino-acid PDB files with *YASARA* (Krieger & Vriend, 2014 ▸), on which *DSSP* was subsequently run to determine the surface accessibility for a fully exposed amino acid.

Additionally, we map data from *HSSP* (Touw *et al.*, 2015 ▸; Sander & Schneider, 1994 ▸) onto the protein sequence. *HSSP* aligns protein sequences from the PDB against UniProtKB and gives the percentage of each amino-acid type at each position in the sequence. This information is less biased than using only sequences from available structure models. It also calculates the Shannon entropy (Shannon, 1948 ▸) per amino-acid position,
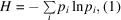
where *H* is the entropy and *p_i_* is the fraction of amino-acid type *i* at a specific position. Both the amino-acid percentage at a position and the Shannon entropy are saved to the database for every residue.

#### 2.2.8. Post-translational modifications   

For each relevant residue, we check whether any of the most prevalent types of post-translational modifications are present. The types of post-translational modifications that are looked for are phosphoryl­ation, glycosylation, methylation, acetylation, carboxylation, hydroxylation, sulfation, oxidation and the cyclization of glutamic acid to pyroglutamate.

Except for glycosylation, this check is based on the residue type. For instance, a phosphorylated threonine should always be called TPO (instead of THR) in PDB entries. All residue types that are searched for are shown in Table 2 ▸. Glycosylation is not based on residue name, because glycosylated residues retain their own residue type and are simply linked to carbohydrate moieties. Hence, if a residue is linked to a carbohydrate residue (which is defined as such in the CCP4 monomer library; Winn *et al.*, 2011 ▸) it is marked as glycosyl­ated.

The presence of a post-translational modification is not only searched for in the structure of interest but also in all homologs. It can then be determined whether a ‘normal’ residue is post-translationally modified in any homologs and also in what percentage of the homologs it occurs. The information on whether a residue is post-translationally modified, whether homologs are modified, the percentage of homologs that are modified and what type of modification occurs is saved to the database. Where multiple types of post-translational modifications are found, it is saved to the database that there is a mixture of types; more exact information will then be available by looking at that residue’s page by clicking the residue.

#### 2.2.9. PDB sequence conservation and ‘orderedness’   

The way in which the mapping of homologous residues between protein structures is performed also immediately provides information on sequence conservation and whether or not homologous residues are ordered (van Beusekom, Joosten *et al.*, 2018 ▸). For each residue, the percentage of homologous residues that have the same residue type, as well as the percentage of homologous residues that are ordered, are stored in the database. This is distinct from the data from *HSSP* as only close homologs that were crystallized are considered.

#### 2.2.10. C^α^ torsion angles   

To compare local conformations of the protein across homologs, C^α^ torsion angles are calculated, *i.e.* the torsion angle for residue *i* is given by the angle across C^α^_*i*−2_, C^α^_*i*−1_, C^α^_*i*_ and C^α^_*i*+1_. The torsion angles of all homologs are then subjected to 1D *k*-means classification as described previously (van Beusekom, Touw *et al.*, 2018 ▸), where the same algorithm was used to obtain targets for hydrogen-bond restraints. Here, it is used to assess whether the local conformation is similar across homologs or whether several classes of conformations are formed. As described above for the determination of Ramachandran angle outliers, angles are transformed prior to classification to deal with torsion-angle periodicity. We also compute whether the structure of interest is in a majority or minority class or whether it is a conformational outlier. This is determined in the same manner as for the Ramachandran outliers described above.

#### 2.2.11. Other model parameters   

Hydrogen bonds are annotated as described previously (van Beusekom, Touw *et al.*, 2018 ▸). The numbers of main-chain and side-chain hydrogen bonds are stored separately in the database.

If a residue has alternate conformations, this information is also stored in the database. A distinction is made between residues with only side-chain alternate conformations and residues that also have alternate conformations of the protein backbone. When alternate conformations are present, the affected distributions, for example rotamers, are not shown in order to avoid the preferential treatment of any particular alternate.

A contact is stored in the database between every residue and ligand pair for which any two atoms are within 3.5 Å of one another. Symmetry is taken into account in computing ligand contacts.

The average *B* factor of all amino acids in the structure model is calculated first. The *B*-factor ratio for a particular amino acid is then computed as the average *B* factor of the atoms of that amino acid divided by the average *B* factor of the whole protein part of the structure model.

If the occupancy of one or more atoms of an amino-acid residue is not equal to 1, the average occupancy is stored in the database.

### 2.3. Website design<!?tpb 0.33pt>   

**Figure 1:**
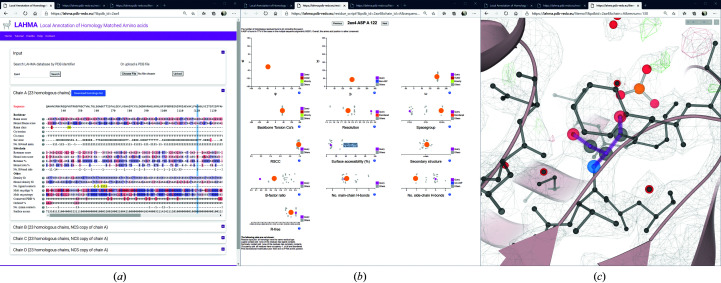
Design of the *LAHMA* website (https://lahma.pdb-redo.eu). (*a*) The front page displays properties of the protein of interest and colors them by the value of the property. NCS copies of the protein chain are hidden by default for clarity. A list of used homologs can be downloaded using the blue button. (*b*) The residue page shows distributions of structural parameters of the protein of interest and its homologs. This page can be accessed by clicking a residue on the front page. The protein of interest is highlighted in magenta. Additional structures can be highlighted (in orange) by mouse-overs. This structure model is then highlighted in all plots. Additional information is given in tooltips. (*c*) Molecular-graphics page for visual inspection of the residues using *LiteMol* (Sehnal *et al.*, 2017 ▸), which can be accessed by clicking a structure model on the residue page.

The website consists of three key pages (Fig. 1 ▸): the entry page, which shows information on all residues in an entry; the residue page, which shows information on a specific residue and all its equivalent amino acids in homologous structure models; and the molecular-graphics page, which centers on a residue in the structure. The residue page can be reached by clicking any residue on the entry page; the molecular-graphics page can be reached by clicking any residue on the residue page. All three pages are described in more detail below.

On the front page (Fig. 1 ▸ *a*), users can either enter a PDB entry identifier or upload a PDB file. The front page is based on the underlying framework of the *Crystallization Construct Designer* 2 (Mooij *et al.*, 2009 ▸; Morris *et al.*, 2019 ▸; https://ccd.rhpc.nki.nl).

Upon entering a PDB identifier, annotated information is presented to the user. This information is color-coded: gliding scales run from blue (good) through white (neutral) to red (bad), outliers are highlighted in red to attract extra user attention and other information of interest is marked in yellow or orange. Details of each metric are given through mouse-overs on question-mark icons. Data lines are not shown when there are no data to describe: for instance, no line on post-translational modifications is shown if none are found in either the structure model or its homologs. A list of used homologs (in JSON format) can be downloaded using the button on the front page (Fig. 1 ▸ *a*).

If a user uploads their own PDB file, computations are run to compare this PDB file with all available homologous data from the PDB-REDO databank. Information from the log file is shown to monitor progress. The duration of the analysis depends on the size of the protein of interest (notably the number of chains) and the number of homologous chains in the databank: coconut allergen cocosin with two chains in the model and five homologous chains in total took 15 s, lysozyme with one chain and 837 homologous chains took 17 s, and influenza B neuraminidase with 16 chains in the model and 78 homologous chains took 21 min. Once the calculation is complete, the page is updated and shows a similar main page as for regular PDB entries. For each uploaded PDB file, a random 32-character code is included in the web address such that a link to the data can be shared among collaborators while limiting others from accessing private information. User-uploaded data are deleted after 48 h.

More information on specific residues of interest can be displayed by clicking them, which opens a new window (Fig. 1 ▸ *b*). This window visualizes the data on that residue and its homologous residues in 18 data categories. For instance, a Ramachandran plot is shown displaying all homologous residues, another plot shows the number of ligand contacts per homologous residue and yet another shows which residues are post-translationally modified. The combination of the plots and mouse-over highlighting allows the user to judge exactly how deviant a residue is. For instance, the ligand plot shows the number of ligand contacts per homologous residue and the mouse-over then shows which ligands these are. Some plots, such as the side-chain torsion-angle plot and the ligand-contact plot, will only be shown if there are data: *i.e.* glycines have no rotamer plot and at least one of the homologous amino acids should have ligand contacts in order for the respective plot to be shown. If a plot is not shown, this is indicated by a message at the bottom of the page. Additional text messages are available that comment on the number of homologous residues and on the conservation in a multiple sequence alignment. Finally, a message is shown if there are any relevant changes between the PDB-REDO and the PDB structure models for this residue, such as a rotamer change or a peptide flip, as this may influence the user’s interpretation of the annotation.

Interesting outliers can be easily and adequately visualized with molecular graphics to inspect them in more detail. This is achieved using the *LiteMol* plugin (Sehnal *et al.*, 2017 ▸) on the molecular-graphics page (Fig. 1 ▸ *c*). Clicking any residue in any plot on the residue page generates a new webpage showing the residue in its structural context, together with the 2*mF*_o_ − *DF*_c_ and *mF*_o_ − *DF*_c_ maps from PDB-REDO at 1.5σ and 3.0σ, respectively.

## 3. Results   

The *annotator* program was run for the entire PDB-REDO databank and the resulting annotation information was stored in the database called LahmaDB. This resulted in over 132 000 entries. Some 2600 PDB-REDO entries were not annotated in LahmaDB because they contained no protein.

The database is kept up to date by automated scripts. When a new or updated PDB-REDO entry is found, a corresponding LahmaDB entry is made. Additionally, existing entries are updated on a weekly basis if the number of homologous chains has increased by at least 10%.

To show the use of *LAHMA*, we first discuss some simple examples and then show two in-depth analyses of proteins that are drug-discovery targets.

### 3.1. Analysis of structural features   

#### 3.1.1. Rotamer outliers   

For each residue, the percentage in which a rotamer (see Section 2.2.4) occurs as well as the total number of rotamers observed at its position are stored in the database. Based on these two numbers, we can easily observe whether a rotamer is an outlier at its position. In the average protein structure model, there are several rotamers that are unusual compared with homologs: part of these are errors and part of these are truly in a different conformation.

**Figure 2:**
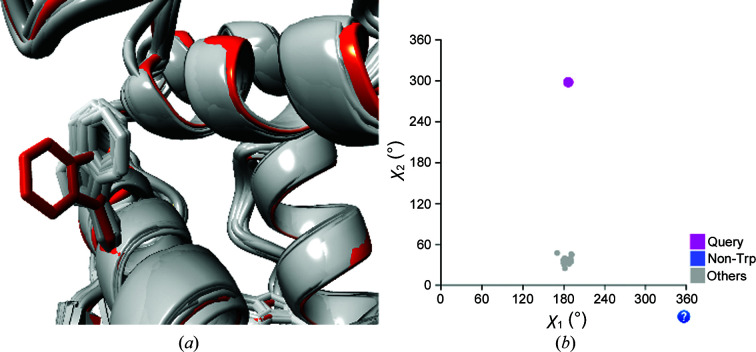
(*a*) A structural alignment of BRCA1 in the area around Trp1782. PDB entry 2ing is shown in red; homologs are shown in gray. Only the Trp1782 side chain is shown; others are left out for clarity. (*b*) Side-chain torsion-angle plot of Trp1782 in all homologous protein structure models; PDB entry 2ing is shown in magenta. All 48 homologs have a χ_1_ angle of around 180° and all but PDB entry 2ing have a χ_2_ angle of around 40°, while PDB entry 2ing has a χ_2_ angle of −63°. Molecular-graphics images, as in the other figures, were made with *CCP*4*mg* (McNicholas *et al.*, 2011 ▸).

In low-resolution models, annotation of homologous information can uncover potentially erroneous features that cannot be observed from the experimental data alone. For instance, in the study of the BRCA1 protein, we observed that Trp1782 in PDB entry 2ing (Tischkowitz *et al.*, 2008 ▸), a 3.6 Å resolution structure model, is found in a different rotamer to all homologous structure models (Fig. 2 ▸). Although the density fit is good (RSCC = 0.93), green difference density appears in the PDB-REDO databank, suggesting another rotamer. It may therefore be worthwhile evaluating cases such as this one in the structure-optimization process to observe whether this rotamer is truly deviant or perhaps is the result of modeling errors. *LAHMA* can be used in this way to improve low-resolution structure models by comparison with their higher resolution homologs.

**Figure 3:**
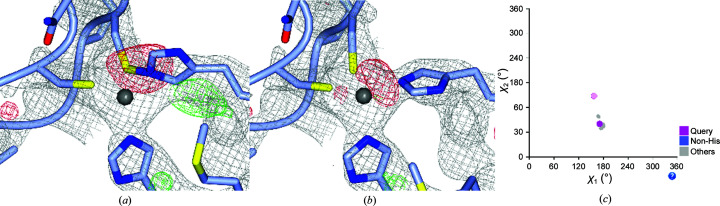
HisA434 was wrongly linked to CysA416 in PDB entry 6blw, prohibiting automated Zn^2+^ site correction in *PDB-REDO*. (*a*) Incorrect PDB-REDO model of PDB entry 6blw. HisA434 is a rotamer outlier with respect to its homologs. The 2*mF*_o_ − *DF*_c_ and *mF*_o_ − *DF*_c_ maps are shown at 1.0σ and 3.0σ, respectively. (*b*) New PDB-REDO model of PDB entry 6blw in which the Zn^2+^ site is corrected and HisA434 is in the same rotamer as its homologs. (*c*) The rotamer torsion angles of HisA434 and all of its homologous histidines. The incorrect conformation of HisA434 is shown in pink (χ_2_ = 150°) and the corrected conformation, which clusters with its homologs, in magenta (χ_2_ = 80°).

A rotamer outlier that is clearly erroneous is found at HisA434 in PDB entry 6blw (Wang *et al.*, 2018 ▸; Fig. 3 ▸). All 20 homologous histidine residues are found in the same rotamer. This histidine is part of a structural Zn^2+^ site, but in PDB entry 6blw it is wrongly linked to CysA416, which is another ligand of the Zn^2+^ ion. Although structural Zn^2+^ sites are automatically corrected by *PDB-REDO* (Touw *et al.*, 2016 ▸), this particular issue had not been observed and therefore was not corrected. The *PDB-REDO* pipeline was updated to deal with this particular type of issue automatically and was used to obtain a more sensible model (Fig. 3 ▸ *b*). χ^2^ was changed from 147° to 80°, which is much more similar to its homologs (Fig. 3 ▸ *c*).

#### 3.1.2. Glycosylation   

**Figure 4:**
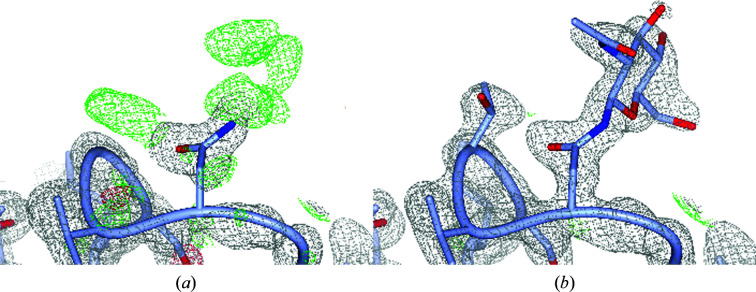
Potential glycosylation site at Asn226 in PDB entry 2oqa. (*a*) The model as deposited in the PDB. (*b*) An improved model in which Gly228 is mutated to threonine and the primary carbohydrate *N*-­acetylglucosamine is modeled, followed by automated optimization using *PDB-REDO*. The 2*mF*_o_ − *DF*_c_ and *mF*_o_ − *DF*_c_ maps are shown at 1.0σ and 2.5σ, respectively.

The presence or absence of post-translational modifications (PTMs) on homologous amino acids could provide valuable new insight. Such an example is found in the structure of luffaculin 1 in PDB entry 2oqa (Hou *et al.*, 2007 ▸). This protein was isolated and crystallized from plant seeds and was sequenced from the 1.4 Å resolution electron-density map. Two *N*-glycosylation sites were discovered by the depositors, but *LAHMA* clearly reveals that Asn226 is also glycosylated in homologs. The local sequence at this asparagine is Asn-Val-Gly, which does not fulfill the common Asn-*X*-Ser/Thr glycosylation-sequence motif (Stanley *et al.*, 2015 ▸). However, a look at the electron density suggests that the glycine may be incorrectly assigned: there is density for a side chain (Fig. 4 ▸ *a*). The multiple sequence alignment from *HSSP* (Touw *et al.*, 2015 ▸) shows that a glycine is only found 2% of the time, while serine and threonine are found in 38% and 15% of cases, respectively, in 213 sequences. Modeling a threonine at position 226 and adding an *N*-acetylglucosamine to Asn226 followed by further model optimization using the *PDB-REDO* web server (Joosten *et al.*, 2014 ▸) removes the difference density and results in a more plausible model (Fig. 4 ▸ *b*).

#### 3.1.3. C^α^ torsion-angle analysis   

By subjecting the C^α^ torsion angles (see Section 2.2.10) from different homologs to *k*-means classification, local conformational outliers can be detected.

**Figure 5:**
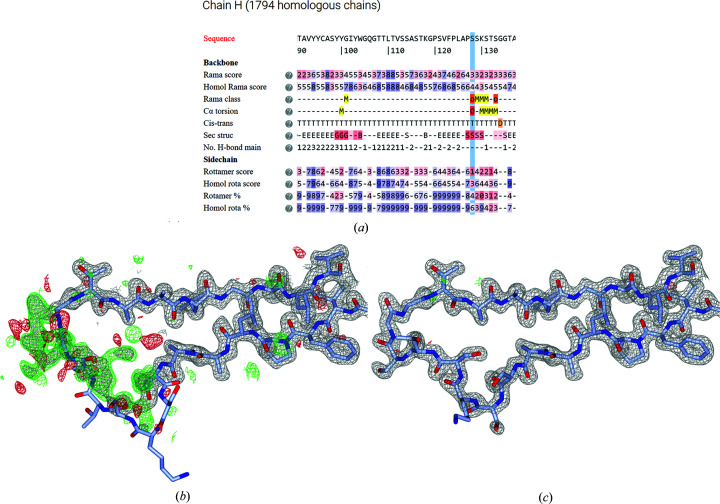
PDB entry 1gaf. (*a*) The area around loop H128–H135 has minority conformations and outliers in Ramachandran plot class and C^α^ torsion angles. (*b*) The PDB-REDO model at present. The current *PDB-REDO* procedure cannot correct the wrongly built loop since it is outside the radius of convergence of (re-)refinement. (*c*) After removing the loop and running *PDB-REDO* with homology-based loop building (van Beusekom, Joosten *et al.*, 2018 ▸), the correct conformation is built with an excellent fit to the electron density. The 2*mF*_o_ − *DF*_c_ and *mF*_o_ − *DF*_c_ maps are shown at 1.5σ and 3.0σ, respectively.

As an example, we look at antibody Fab fragments, which are some of the most common proteins in the PDB (nearly 1800 homologs). In PDB entry 1gaf (Patten *et al.*, 1996 ▸) the C^α^ torsion angle is an outlier for residue H128 and a minority conformation for residues H130–H133: a clear indication that something is abnormal compared with its homologs (Fig. 5 ▸ *a*). When inspecting the density, it appeared that the loop between H128 and H135 is completely pulled out of its electron density (Figs. 5 ▸ *b* and 5 ▸ *c*), which is likely to be caused by the use of simulated annealing in model refinement (Patten *et al.*, 1996 ▸). The other areas in this antibody singled out as ‘different’ from homologs are some of the residues in the complementarity-determining regions and those immediately adjacent, which is to be expected in antibody fragments.

### 3.2. Use cases for analysis of protein families   

#### 3.2.1. Autotaxin   

Autotaxin (ATX or ENPP2) is a secreted glycoprotein that hydrolyses lysophosphatidylcholine into lysophosphatidic acid (LPA) and choline, and is a well established target for several pathologies, including a phase III clinical trial for treating idiopathic lung fibrosis.

ATX has been experimentally shown to contain three N-linked glycosylation sites (Jansen *et al.*, 2007 ▸). Of these, Asn524 was shown to be essential for ATX activity, whereas Asn53 and Asn410 have commonly been mutated to alanine residues to facilitate the crystallization process. Therefore, we first used *LAHMA* to examine the glycosylation states of all ATX structures. In *LAHMA*, this can be performed directly without having to check all 43 ATX structures separately; one needs to inspect just one of the homologs. Asn524 was bound to an N-linked glycan in all cases, which confirms the literature reports. On the other hand, residues 53 and 410 were found to be either alanine or asparagine, depending on the structure. Interestingly, the wild-type Asn residues were found to be both glycosylated and nonglycosylated in different structures, which confirms that this is not a necessary post-translational modification for folding and/or activity.

**Figure 6:**
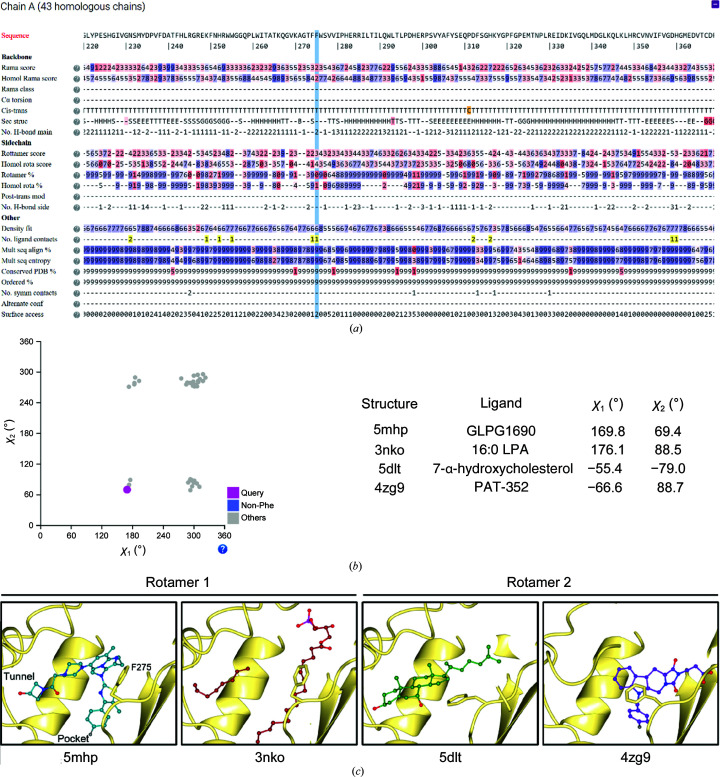
(*a*) Screenshot showing a search for PDB entry 5mhp on the* LAHMA* website, where a poor Ramachandran plot score on Phe275 can be seen (highlighted in blue). (*b*) The distribution of χ_1_ and χ_2_ side-chain torsion angles of 43 homologous structures, where four clusters can be seen. However, because 180° rotations around χ_2_ are chemically equivalent, just two real clusters varying in χ_1_ only are distinguished. (*c*) Crystal structures showing that rotamer 1 (χ_1_ ≃ 180°) occurs when both the tunnel (left) and the pocket (bottom) bind ligands (PDB entries 5mhp and 3nko), whereas rotamer 2 (χ_1_ ≃ −60°) mainly appears when only one site is occupied (PDB entries 5dlt and 4zg9).

The compound currently in phase III clinical trials is GLPG1690; this drug candidate spans the hydrophobic lipid-binding pocket and the adjacent partially hydrophobic allo­steric site (the tunnel). Using *LAHMA*, we explored the crystal structure containing GLPG1690 (PDB entry 5mhp; Desroy *et al.*, 2017 ▸). This showed that Phe275, which is close to the catalytic site, has poor Ramachandran and rotamer plot scores (Fig. 6 ▸ *a*). Further inspection of this residue showed that it was segregated into four separate side-chain conformations depending on the χ angles of the side chain, namely χ_1_ ≃ 180° or 300° (−60°) and χ_2_ ≃ 80° or 280° (−80°) (Fig. 6 ▸ *b*). Since the two χ_2_ angles represent a 180° rotation of the phenyl group in Phe275, Phe275 exists in two distinct conformation groups with χ_1_ ≃ 180° and χ_1_ ≃ −60°. Phe275 in PDB entry 5mhp belongs to the first rotamer group, creating aromatic ring stacking between GLPG1690 and Phe275 (Fig. 6 ▸ *c*). These hydrophobic interactions are also observed with a related drug (PDB entry 5m7m; Joncour *et al.*, 2017 ▸) and with two compounds occupying the hydrophobic pocket in PDB entries 5ohi and 5olb (Kuttruff *et al.*, 2017 ▸). Furthermore, this rotamer also occurred when the product of the protein, LPA, was bound in the tunnel, for example in PDB entries 3nkn, 3nko and 3nkr (Nishimasu *et al.*, 2011 ▸). Conversely, the second rotamer group (χ_1_ ≃ −60) includes structures that contain a chemically different series of tunnel-and-pocket-spanning compounds with which Phe275 is not able to establish aromatic stacking, structures in which a ligand is only bound in the tunnel (for example 5JK in PDB entry 5dlt; Keune *et al.*, 2016 ▸) or in the pocket (4O2 in PDB entry 4zg9; Stein *et al.*, 2015 ▸) or ligand-free structures. In this second conformer group, Phe275 appears to be blocking the entrance to the tunnel (Fig. 6 ▸ *c*). In this case, we show how *LAHMA* helps in the conclusion that Phe275 can act as a rail switch depending on the binding sites that a specific compound occupies.

#### 3.2.2. MPS1 kinase   

The human protein monopolar spindle 1 (Mps1) kinase is a master regulator of the mitotic spindle-assembly checkpoint, ensuring faithful chromosome segregation during mitosis (Sacristan & Kops, 2015 ▸). Owing to its role in the viability of tumor development, Mps1 kinase is an attractive target for oncological drug discovery (Liu & Winey, 2012 ▸; Pachis & Kops, 2018 ▸). To date, more than 60 crystal structures of the Mps1 kinase domain, all in complex with various inhibitors or cofactors, have been deposited in the PDB (Roorda *et al.*, 2019 ▸).

**Figure 7:**
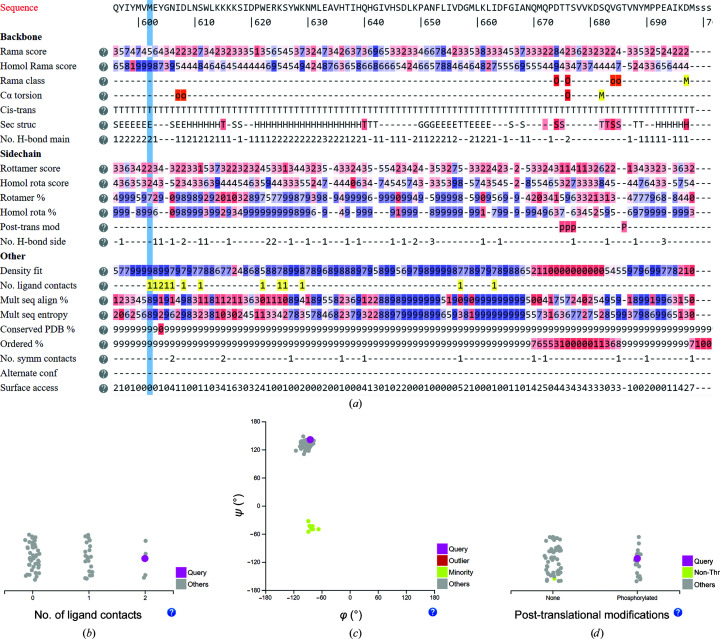
*LAHMA* output for Mps1 kinase with PDB entry 5ntt as the query structure. (*a*) Notable structural features are clearly highlighted: residues 602–606 contact the ligand, residue 686 is phosphorylated, residues 675–677 that are part of the activation loop of Mps1 kinase are found to be phosphorylated in homologs but not in this structure model, and the activation loop is typically disordered in crystal structures of Mps1 kinase. (*b*) Number of ligand contacts for residue 604. In about half the cases this residue makes one or more ligand contacts. (*c*) The Ramachandran plot distribution for residue 604 shows two distinct clusters. (*d*) Phosphorylation of residue 686 is found in a quarter of the structure models.

Examining all Mps1 kinase family structures in *LAHMA* (Fig. 7 ▸ *a*) easily highlights a ‘yellow’ stretch of ligand contacts at residues 602–606: this draws attention to this region as being crucial in ligand binding. Indeed, it is well established in the literature that these so-called ‘hinge-region’ residues form hydrogen bonds to the inhibitor in all reported structures of Mps1 kinase (Fig. 7 ▸ *b*). Additionally, it is obvious that Cys604 (red) is not conserved in the PDB structures. Clicking this residue and inspecting the residue-type plot shows that this residue is commonly a cysteine but is sometimes a tryptophan. This again easily points to previous studies, which show that a point mutation at Cys604 of the hinge region to tyrosine or tryptophan raises resistance against a number of inhibitors, including Cpd-5 as well as NMS-P715, but not against the well characterized Mps1 kinase inhibitor reversine (Koch *et al.*, 2016 ▸; Hiruma *et al.*, 2017 ▸).

A closer look at Cys604 in the residue panel for residue 604 (Fig. 7 ▸ *b*) shows that while about half of the inhibitors make contacts with this residue, others do not. Interestingly, all tryptophan and tyrosine mutants of Cys604 (PDB entries 5mrb and 5ntt, 5ljj for the Tyr mutation and PDB entries 5o91, 5ap6 and 5ap7 for the Trp mutation; Hiruma *et al.*, 2016 ▸, 2017 ▸; Gurden *et al.*, 2015 ▸) make at least one contact with the inhibitor bound in the active site, but inhibitors in structures with the native cysteine in most (but not all) cases make no contacts. This could provide insight for the rational design of novel inhibitors that would not lead to resistance or could be used when resistance arises in specific tumors.

Interestingly, the Ramachandran plot for residue 604 shows two distinct clusters with the ψ angle differing by 180° (Fig. 7 ▸ *c*). The minority cluster contains only cysteine residues, whereas the majority cluster also contains tyrosine and tryptophan mutants. There are no clear correlations with other structural parameters, but visual inspection shows that the peptide bond between Cys604 and Gly605 has reoriented to accommodate ligands with a hydrogen-bond donor that interacts with the carbonyl O atom of Cys604. Most of these ligands have an anilino-pyridine scaffold (Kusakabe *et al.*, 2012 ▸).

Other interesting insights into the Mps1 kinase family are found by analyzing the phosphorylation states of the P1 and P2 loops. *LAHMA* quickly shows which amino acids are phosphorylated in which structure models: it is trivial to spot residues Thr675, Thr676, Ser677 and Thr686 in *LAHMA* (Fig. 7 ▸ *a*), which are well known autophosphorylation sites that affect Mps1 kinase activity (Liu & Winey, 2012 ▸). Approximately a quarter of the reported structure models show phosphorylation on Thr686 (Fig. 7 ▸ *d*). There is much less structural information on Thr675 and Thr676, as these residues are not commonly ordered (Fig. 7 ▸ *a*): ten and seven cases out of 69 are ordered. Only four of these are phosphorylated on Thr675 [PDB entries 5ap1 (Gurden *et al.*, 2015 ▸), 3h9f (Kwiatkowski *et al.*, 2010 ▸), 4o6l and 4js8 (W. Qiu, A. N. Plotnikov, O. Plotnikova, M. Feher, D. E. Awrey & N. Y. Chirgadze, unpublished work)]. Thr676 and Ser677 are ordered and phosphorylated in the first three structure models, but in PDB entry 4js8 they are not ordered so the phosphorylation state cannot be inferred from this crystal structure. In PDB entries 5ap1, 3h9f and 4js8 the activation loop contacts a symmetry copy of itself, which does not occur in the non­phosphorylated activation loops. This correlation can be clearly seen by highlighting individual entries in the post-translational modification and number of symmetry contact plots.

In addition, the residues in PDB entries 5ap1 and 3h9f are marked as making ligand contacts with magnesium. Visual inspection confirms that the magnesium ions stabilize the conformations of the phosphorylated residues. We conclude that order of residues Thr676 and Ser677 can be induced by using the right crystallization conditions: PDB entries 5ap1 and 3h9f were obtained from crystallization with 0.2 *M* magnesium chloride, whereas PDB entry 4js8 was obtained from crystallization with 0.2 *M* ammonium sulfate.

Phosphorylation of the activation-loop residues (Thr675–Ser677) is typically a priming event for canonical kinase activation (Liu & Winey, 2012 ▸). Interestingly, however, all of the crystal structures of Mps1 kinase, even those with phosphorylated activation loops, adopt an inactive conformation (Roorda *et al.*, 2019 ▸). The structural role of the phosphorylation remains to be elucidated.

We thus show how *LAHMA* can be used to easily point out existing insights into the Mps1 kinase family and also how new insights can emerge by careful examination of the data.

## 4. Discussion and conclusions   

The *LAHMA* server presented here can be used for more complete interpretation of protein structures and for the improvement of protein structure models. Users are enabled to observe within minutes (or even seconds) those features that make their structure model of interest unique compared with its structural homologs. Usually, this requires a process of manual structure selection, structural superposition and then manual inspection of the data, which is not only much more work but is also likely to be incomplete since a complete manual inspection requires much time and experience.

In future applications, systematic comparison of a structure with all available homologs will be useful for better automated structure-model optimization. For instance, correcting from *trans* to *cis* is very computationally intensive to attempt for each amino acid. Given the very low frequency of occurrence of *cis*-peptides and the risk of false positives, such a brute-force approach is not sensible. After comparing homologs, peptide bonds that vary in state across homologs can now easily be found. These are far fewer and thus can be scrutinized in great detail. Similarly, if we can automatically detect the areas in a protein structure model that fit the density poorly and are conformational outliers compared with their homologs at the same time, these areas could be automatically refitted. We have already shown an example of this: the poorly fitted loop in PDB entry 1gaf discussed above (Fig. 5 ▸) was manually removed but was added back in the correct conformation using a recently published homology-based loop-building methodology (van Beusekom, Joosten *et al.*, 2018 ▸).

The *LAHMA* server is unique in that while it does not use sequence conservation but protein structure to analyze and annotate proteins in the context of their homologs, it does so without performing a structural superposition in three-dimensional space. This approach is complementary to other protein-annotation systems such as *PDBe-KB* (Varadi *et al.*, 2020 ▸) and 3*D-Bionotes* (Segura *et al.*, 2017 ▸).

The setup of *LAHMA* is modular, which allows further expansion of the described feature set. A possible future extension is to compute correlations between different residues. For instance, correlated motion is implicated if several residues are consistently found in a minority rotamer in a subgroup of the homologous structure models or if they correlate in whether or not they have alternate conformations. Such an analysis would separate homologs into structural subgroups that can be compared to find correlations with biochemical states such as bound ligands or complex formation.

The *LAHMA* server can be used to uncover errors as well as interesting features of protein structure models, as has been shown with many examples. With little effort, researchers will find sites that warrant further study in their proteins of interest.

## 5. Availability   

The *LAHMA* website is available at https://lahma.pdb-redo.eu and is open to all users without restrictions. Lists of detected homologs are available from the PDB-REDO databank at https://pdb-redo.eu/db/####/####_available_homologs.json, with #### denoting the PDB identifier in lower case letters.

## Supplementary Material

Supplementary Figure S1. DOI: 10.1107/S2059798320014473/ir5018sup1.pdf

## Figures and Tables

**Table 1:** Overview of the model parameters stored in *LAHMA*’s database See Section 2.2 for details of the computation of these parameters.

Protein structure feature	Parameters stored in database
Backbone conformation	
Homology-independent	φ, ψ, ω torsion angles; C^α^ torsion angles†; Ramachandran *Z*-­score; number of hydrogen bonds; secondary structure
Relative to homologs	Relative Ramachandran *Z*-score‡; Ramachandran outliers; *cis*/*trans* outliers; C^α^ torsion-angle outliers; percentage of homologous residues with the same secondary structure
Side-chain conformation	
Homology-independent	χ_1_–χ_4_ torsion angles; rotamer *Z*-score; number of hydrogen bonds
Relative to homologs	Relative rotamer *Z*-score‡; percentage of homologs in the same rotamer; number of different rotamers at this position
Experimental data fit metrics	
Homology-independent	RSCC, RSCC *Z*-score, *R*_free_
Relative to homologs	Relative RSCC *Z*-score‡
Post-translational modifications	
Homology-independent	The modification of the residue
Relative to homologs	Whether any homologous residues are modified and, if so, the percentage and type of the modification
Others	
Homology-independent	One-letter code; number of symmetry contacts; presence of alternate conformations of the residue; relative surface accessibility; ligand contacts; *B*-factor ratio relative to the mean; average occupancy
Relative to homologs	Residue conservation compared with the PDB and *HSSP* §; percentage of homologous residues that is ordered in the PDB; sequence entropy from *HSSP*

† The torsion angle over four sequential C^α^ atoms as a means of measuring local conformation.

‡ A *Z*-score of *Z*-­scores, which compares the *Z*-score of the residue of interest with the *Z*-scores of homologous amino acids.

§ See Section 2.2.7.

**Table 2:** Detected post-translational modifications PDB residue names of recognized modified amino acids with their corresponding amino-acid base type, ordered by the type of post-translational modification (PTM). Glycosylation is not included in this table (see Section 2.2.8).

PTM type	Modified amino acids	Standard amino acids
Phosphorylation	TPO, SEP, PTR, NEP, HIP	THR, SER, TYR, HIS, HIS
Methylation	M3L, MLY, MLZ, 2MR, HIC, MHS, SMC	LYS, LYS, LYS, ARG, HIS, HIS, CYS
Acetylation	ALY, OAS	LYS, SER
Carboxylation	KCX, CGU	LYS, GLU
Hydroxylation	HYP, 0AF	PRO, TRP
Sulfation	TYS	TYR
Oxidation	CSD, CSO, OCS, SME, MHO, OMT	CYS, CYS, CYS, CYS, MET, MET
Pyroglutamate	PCA	GLU
