# Supplementary material for: LAHMA: structure analysis through local annotation of homology-matched amino acids
Source: Acta Crystallogr D Struct Biol. 2021 Jan 1;77(Pt 1):28–40. doi: 10.1107/S2059798320014473 (PMC7787103; doi:10.1107/S2059798320014473)
Supplement: Supplementary file 1 [file d-77-00028-sup1.pdf]

STRUCTURAL
BIOLOGY

Volume 76 (2020)

Supporting information for article:

LAHMA: structure analysis through local annotation of homology-matched amino acids

Bart van Beusekom, George Damaskos, Maarten L. Hekkelman, Fernando Salgado Polo, Yoshitaka Hiruma, Anastassis Perrakis and Robbie P. Joosten

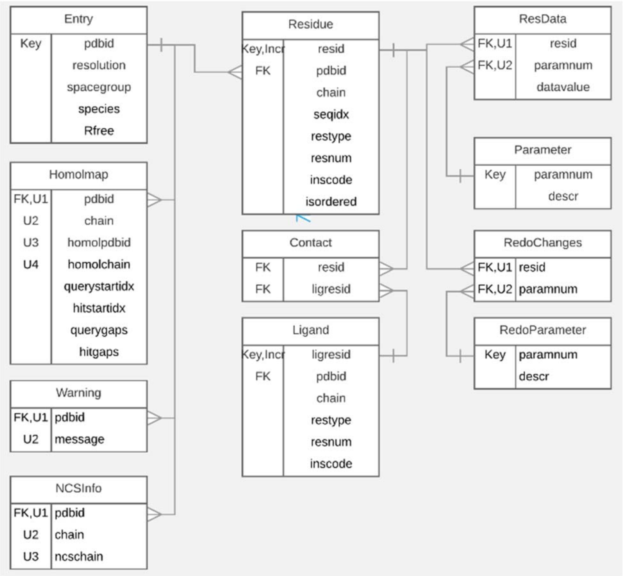

Figure S1 Scheme of the database. The following information describes the tables: key, primary key of the table; FK, foreign key in a different table; Incr, this variable is auto-incremented; U1, U2, etc., parameters that together are constrained to be unique.
